# Development of Strand-Specific Real-Time RT-PCR to Distinguish Viral RNAs during Newcastle Disease Virus Infection

**DOI:** 10.1155/2014/934851

**Published:** 2014-10-14

**Authors:** Xusheng Qiu, Yang Yu, Shengqing Yu, Yuan Zhan, Nana Wei, Cuiping Song, Yingjie Sun, Lei Tan, Chan Ding

**Affiliations:** Shanghai Veterinary Research Institute, Chinese Academy of Agricultural Sciences (CAAS), 518 Ziyue Road, Shanghai 200241, China

## Abstract

Newcastle disease virus (NDV) causes large losses in the global fowl industry. To better understand NDV replication and transcription cycle, quantitative detection methods for distinguishing NDV genomic RNA (gRNA), antigenomic RNA (cRNA), and messenger RNA (mRNA) in NDV-infected cells are indispensible. Three reverse transcription primers were designed to specifically target the nucleoprotein (NP) region of gRNA, cRNA, and NP mRNA, and a corresponding real-time RT-PCR assay was developed to simultaneously quantify the three types of RNAs in NDV-infected cells. This method showed very good specificity, sensitivity, and reproducibility. The detection range of the assay was between 5.5 × 10^2^ and 1.1 × 10^9^ copies/*μ*L of the target gene. These methods were applied to investigate the dynamics of the gRNA, cRNA, and mRNA synthesis in NDV La Sota infected DF-1 cells. The results showed that the copy numbers of viral gRNA, cRNA, and NP mRNA all exponentially increased in the beginning. The viral RNA copy number then plateaued at 10'h postinfection and gradually decreased from 16 h postinfection. No synthesis priority was observed between replication (gRNA and cRNA amounts) and transcription (mRNA amounts) during NDV infection. However, the cRNA accumulated more rapidly than gRNA, as the cRNA copy number was three- to tenfold higher than gRNA starting from 2 h postinfection. *Conclusion*. A real-time RT-PCR for absolute quantitation of specific viral RNA fragments in NDV-infected cells was developed for the first time. The development of this assay will be helpful for further studies on the pathogenesis and control strategies of NDV.

## 1. Introduction

Newcastle disease virus (NDV) is an economically devastating pathogen in the global poultry industry [1, 2]. NDV, a member of the family Paramyxoviridae (genus* Avulavirus* in subfamily Paramyxovirinae) [3], has a single-stranded, nonsegmented negative-sense RNA genome of approximately 15 kb. Six viral structural proteins are encoded from its genome: a nucleoprotein (NP), phosphoprotein (P), matrix protein (M), fusion protein (F), haemagglutinin-neuraminidase (HN), and large protein (L) [4–7]. In addition to those viral gene products, two more nonstructural proteins, V and W, are encoded from the P gene via an RNA-editing mechanism [8–10].

All NDV viral mRNAs are transcribed from NDV genomic RNA by using the viral RNA-dependent RNA polymerase (vRdRp) assembled from NP, P, and L proteins [11, 12]. The L protein is the core element of vRdRp, containing the polymerase activity as well as capping and polyadenylation activities [13–17]. The P protein acts as a bridge between the L protein and NP protein which encapsidates the viral genome [18]. The NP-P complex is thought to be the substrate used by the RdRp to initiate the encapsidation of the nascent RNA chain during viral replication [19, 20].

In the early phase of NDV infection, the HN protein of NDV attached to the surface of cells and the following fusion between viral and cell membranes was mediated by the F protein [21]. Subsequently, the NP-wrapped viral genomic RNA, as well as other viral components, was released into the cytoplasm [22, 23]. The NP protein encapsidates viral gRNA and cRNA to resist host nucleases and also mediates viral RNA replication and transcription by RdRp [12, 24, 25]. The (−) viral genomic RNA, termed gRNA, is a template for synthesis of mRNA and the intermediate (+) viral genomic RNA (termed cRNA in this study); the latter was used as the template for gRNA synthesis [7, 26, 27]. The cRNAs serve as replicative intermediates for gRNA duplication and are not known to encode viral proteins [28].

The amounts of viral gRNA, cRNA, and mRNA reportedly change in a regular pattern in cells infected with the measles virus, influenza virus, and Nipah virus [29–32], suggesting these RNA viruses regulate viral replication and transcription through specific molecular mechanisms. In this study, a strand-specific real-time RT-PCR assay was developed to distinguish viral gRNA, cRNA, and mRNA levels in NDV-infected cells. This will aid studies into the molecular mechanisms of viral replication and transcription, viral pathogenesis, and control strategies for NDV.

## 2. Materials and Methods

### 2.1. Cell and Virus

DF-1 cells were obtained from the American Type Culture Collection (ATCC, Manassas, VA, USA) and grown in RPMI-1640 media (10% fetal bovine serum, 2 mM L-glutamine, 100 IU/mL penicillin, 100 *μ*g/mL streptomycin, and 10 mM HEPES buffer) at 37°C and 5% CO_2_. Cell culture reagents were obtained from Gibco (Rockville, MD, USA).

NDV La Sota strain was obtained from the China Institute of Veterinary Drug Control (Beijing, China) and propagated in 9- to 11-day-old specific-pathogen-free (SPF) embryos. The infected allantoic fluid was harvested between 84 and 120 h. The virus was determined for the 50% tissue culture infective dose (TCID_50_) and kept at −80°C before use.

### 2.2. Virus Infection

The incubation time for the virus infection was optimized first. DF1 cells (3 × 10^5^ cells) were cultivated in 60 mm dishes at 37°C and 5% CO_2_. For La Sota infection, a monolayer of DF1 cells was washed with phosphate-buffered saline (PBS) three times and 1 mL of serum-free 1640 medium containing NDV La Sota at a multiplicity of infection (MOI) of 0.01 was then added. At 5 s, 1 min, 5 min, 10 min, 15 min, 20 min, 25 min, 30 min, and 60 min postinfection (pi), the cells were washed again with PBS to remove unbound virus particles and harvested for RNA extraction and subjected to real-time RT-PCR. The optimal incubation time for NDV infection was based on when the cDNAs of gRNA, cRNA, and mRNA of NP started to stably accumulate. The quantitation cycle (*C*_*q*_) values of each RNA at this time point were considered residual viral RNAs after incubation and set as baseline values. According to the optimized incubation time determined, DF1 cells were infected with La Sota at an MOI of 0.01. After incubation, cells were washed with PBS and then grown in 4 mL 1640 medium containing 1% (v/v) fetal calf serum at 37°C and 5% CO_2_. The cells and supernatant were harvested at different time points for the assay.

### 2.3. RNA Extraction and Strand-Specific Reverse Transcription

After infection with NDV La Sota, the supernatant and cells were collected and thoroughly mixed and 500 *μ*L of the mixture was lysed with 1 mL TRIZol reagent (Invitrogen, Carlsbad, CA, USA). The total RNA of the virus was extracted according to the manufacturer's instructions. The RNA pellets were resuspended in 50 *μ*L RNase-free water after washing with 70% ethanol. To degrade cellular DNA, DNase* I* was added to the samples at a final concentration of 1 U/*μ*L at 37°C for 30 min and then inactivated at 65°C for 10 min in the presence of 5 mM EDTA.

**Figure 1:**
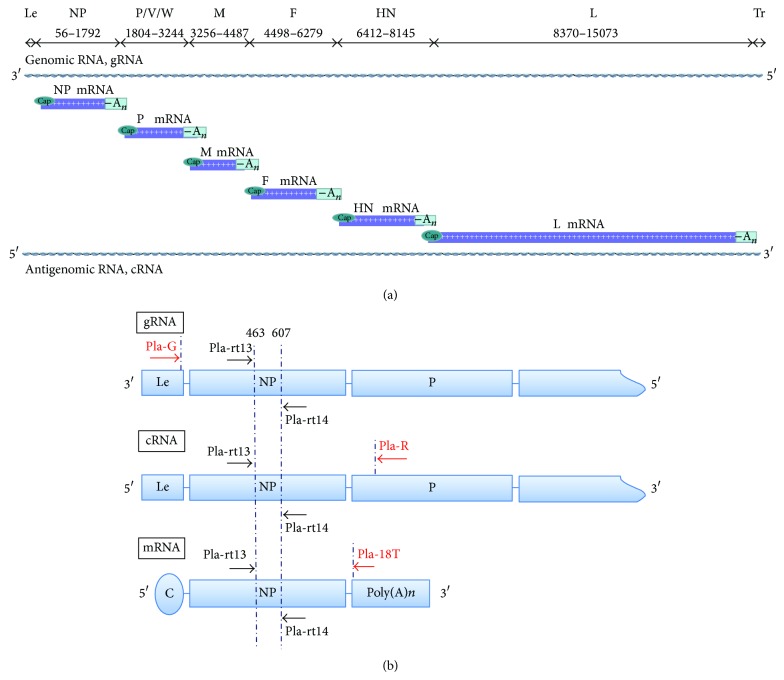
Schematic chart of primer design. (a) Schematic view of NDV genomic organization, main RNA species, and nucleotide positions. (b) Binding position of RT-qPCR primers. Red arrows represent the locations of primers for reverse transcription. Black arrows represent the locations of primers for real-time RT-PCR amplification.

Strand-specific reverse transcription of NDV gRNA, cRNA, and mRNA was performed with specifically designed reverse transcription primer sets (Figure 1). To quantify the three kinds of cDNAs with one pair of primers, reverse transcription primers specific for gRNA, cRNA, or mRNA were designed using the Lasergene software suite (DNASTAR, Madison, WI, USA).

The sequence of the gRNA-specific reverse transcription primer PLa-G was 5′-ACG ATA AAA GGC GAA GGA GCA-3′ (genomic position 22–44 nt), which initiated synthesis of the unique leader sequence of the NDV genome.

The sequence of primer PLa-R specific to the P gene of viral cRNA was 5′-GTC GTT TGC TCG GGT GTG GAT G-3′ (genomic position 2, 119-2, 139 nt).

The extracted mRNA segments, involving viral mRNA, were reverse-transcribed by PLa-18V (5′-TTT TTT TTT TTT TTT TTT A/G/C-3′).

Each 10 *μ*L of the above RNA samples was reverse-transcribed by respective primers in the presence of M-MLV reverse transcriptase (Promega, Madison, WI, USA), according to the protocol. Briefly, 200 U M-MLV reverse transcriptase, 5 *μ*L 5 × RT buffer, 10 *μ*L RNA sample, 10 mmol specific RT primer, 10 mmol dNTPs, and ultrapure water (Gibco BRL, Gaithersburg, MD, USA) were added to a final volume of 25 *μ*L and mixed. The mixture was incubated at 42°C for 60 min and then 75°C for 15 min to denature reverse transcriptase. The reverse transcripts were precipitated using two volumes of ethanol. Finally, the yielded pellet was dissolved in 20 *μ*L of ultrapure water (Gibco BRL) and kept at −70°C until use.

### 2.4. Real-Time Quantitative Polymerase Chain Reaction (PCR) Assay

#### 2.4.1. Construction of Plasmid T-NP as a Standard for the Assay

To construct a standard curve for the linear correlation between *C*_*q*_ values and molecular numbers of target cDNAs, plasmid T-NP was constructed and used as a standard. Genomic RNA was reverse-transcribed by random hexamer primers, and 5 *μ*L of the resultant cDNA fragments was then added into a 50 *μ*L PCR reaction system containing 2.5 U TaKaRa LA* Taq*, 5 *μ*L 10 × LA PCR Buffer II (Mg^2+^ Plus), 1 *μ*L 20 *μ*M former primer PNPF, 1 *μ*L 20 *μ*M reverse transcription- (RT-) primer PNPR, and 8 *μ*L 2.5 mM dNTP mixture. The primer pairs of PNPF (5′-CAG TGA TGA CCC AGA AGA TAG ATG-3′) and PNPR (5′-CGC AAA GCT CAT CTG GTC ACT ATC-3′) were used to amplify the NP gene. The PCR cycle conditions were 95°C 1 min, 25 cycles of 94°C 30 s, 55°C 30 s, and 72°C 90 s, followed by 72°C 10 min.

The PCR product was subcloned into the pGEM-T easy vector (Promega) to construct the plasmid T-NP and identified by nucleotide sequence analysis. Plasmid DNA was purified using QIAGEN Plasmid Midi Kits, and then the concentration and purity were determined by the ND-1000 spectrophotometer (NanoDrop Technologies, Inc.). The purified plasmid T-NP with an A260/280 ratio of 1.80 qualified as a standard.

#### 2.4.2. Optimization of the Real-Time Quantitative PCR Procedure

To obtain suitable amplification efficiency, three pairs of primers, including Pla-rt11/Pla-rt12, Pla-rt13/Pla-rt14, and Pla-rt21/Pla-rt22 pairs (Table 1), were designed according to “*MIQE guidelines for PCR*” [33, 34], based on the cDNA sequence of the NDV La Sota strain (GenBank ID: LaSota_AF077761), and used for NP gene amplification with viral cDNA and T-NP as templates. Following the manufacturer's instructions, quantitative PCR (qPCR) was performed using Maxima SYBR Green dye (Fermentas, Glen Burnie, MD, USA) in a PCR thermocycler from Eppendorf AG (Hamburg, Germany) operated by the Mastercycler ep realplex system. The qPCR reaction mixture comprised 12.5 *μ*L of 2x Maxima SYBR Green, qPCR Master Mix, 1.0 *μ*L of reverse transcription product, and 25 pmol of each real-time primer, made up to a final volume of 25 *μ*L with ultrapure water (Gibco BRL). The cycle conditions of qPCR were 95°C 5 min, followed by 40 cycles of 95°C 15 s, 60°C 30 s, and 72°C 30 s. Nuclease-free ultrapure water (Gibco BRL) was used as a template for the negative control. The specificity of the qPCR primers was monitored with melting curve analysis.

#### 2.4.3. Linear Regression Analysis

To establish the statistical relationship between *C*_*q*_ values and the molecular numbers of samples, 5 ng/*μ*L T-NP plasmid (A260/280 ratio = 1.80) was fivefold serially diluted and quantitated with the primer pairs of Pla-rt13 and Pla-rt14. A standard curve was then generated by plotting *C*_*q*_ values against logarithmic molecular numbers of the standard, and linear regression analysis was conducted to obtain a linear regression equation.

#### 2.4.4. Specificity of the Assay

To determine the specificity of the Pla-rt13 and Pla-rt14 primers, RT-PCR was performed with TaKaRa LA Taq to obtain DNA fragments comprising portions of the viral genome other than the NP gene. Eight primer pairs (Table 1) targeting genes other than NP were designed using the Lasergene software suite (DNASTAR) and used for PCR amplification of NDV genes other than NP.

The PCR products were purified, and their concentration and purity were tested using a ND-1000 spectrophotometer (NanoDrop Technologies, Inc.). Only the PCR products with an A260/280 ratio of 1.80 qualified for the study. The eight PCR products were tenfold serially diluted and quantified. The *C*_*q*_ values of PCR products with different concentrations were compared with that of T-NP and the negative control (nuclease-free ultrapure water).

#### 2.4.5. Sensitivity and Reproducibility of the Assay

To confirm assay sensitivity and reproducibility, the T-NP plasmid (5 ng/*μ*L) was tenfold serially diluted to 0.0005 ng/*μ*L and used for the detection. The amplification efficiency and coefficient of variation (CV) of the qPCR reaction were compared between different template concentrations, and the detection range was determined. The experiment was performed in triplicate.

#### 2.4.6. Quantitative Analysis of Viral RNA Kinetics

Total viral RNAs extracted from NDV-infected cells at 1, 2, 3, 4, 5, 6, 8, 10, 12, 16, 20, 24, 36, and 48 hpi were quantitatively analyzed. The samples were first reverse-transcribed with primers PLa-G, PLa-R, or PT-18V. The resultant cDNA products (1 *μ*L cDNA product was used for each reaction) were then quantified with the primer pairs of Pla-rt13 and Pla-rt14. The total RNA of the DF-1 cells was used as a negative control. Considering the interference of other ingredients in each sample, the absorption of cellular RNA harvested just after 20 min was also detected and set as base line. The values obtained by the real-time RT-PCR assay were analyzed by linear regression and the kinetic curves of gRNA, cRNA, and mRNA in La Sota-infected DF1 cells were generated.

## 3. Results

### 3.1. Establishment and Validation of a Real-Time RT-qPCR Assay for NDV

Three pairs of primers, including Pla-rt11/Pla-rt12, Pla-rt13/Pla-rt14, and Pla-rt21/Pla-rt22 pairs (Table 1), were initially designed and used for the amplification. Neither nonspecific annealing nor primer dimer was observed in the melting curves of these three primer pairs. Pla-rt13/Pla-rt14 displayed an extremely stable amplification efficiency around 1.000, while those of Pla-rt11/12 and Pla-rt 21/22 were 0.981 and 0.987, respectively. In this study, the Pla-rt13/Pla-rt14 primer pairs were used for accurate qPCR amplification (Figure 1(a)).

Eight PCR fragments, Pla1–Pla8, which comprised most of the NDV genomic sequence, were obtained at the expected sizes. After sequencing, these fragments were confirmed and then primed with Pla-rt13 and Pla-rt14 in a real-time PCR reaction. No significant cross-activity was detected with those DNA fragments. No cross-activity was shown with RNA extracts from uninfected cells or normal SPF chicken embryo allantoic fluid. Thus, the developed qPCR assay demonstrated excellent specificity.

For quantitating cDNA molecules, a standard curve was generated with a fivefold serial dilution of the T-NP plasmid. The amplification efficiency was 1.01 and the concentration at which linearity was retained in the standard curve was in the range of 5.5 × 10^2^–1.1 × 10^9^ copies/*μ*L (the detection range of the assay). Using linear regression, a partial regression line was calculated and the linear regression equation was *Y* = −3.290, 1*X* + 38.922 (Figure 1(b)). An *R* ^2^ value of 0.998 indicated strong linear correlations. The highest *C*_*q*_ value of tested NTC (in this case, ultrapure water was used for template dilution) was 31.8; therefore, test results with a *C*_*q*_ value lower than 26.80 were considered positive.

**Figure 2:**
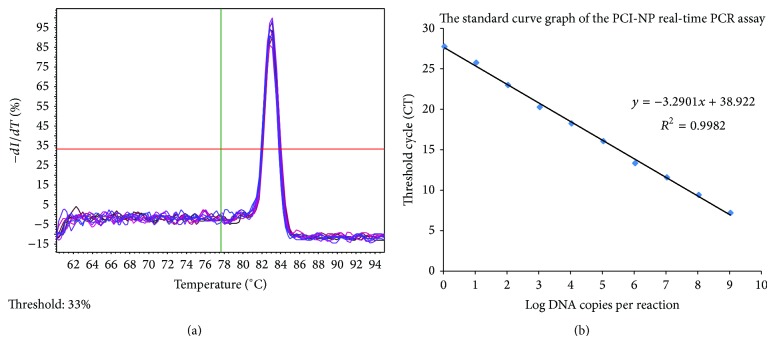
The development of a real-time quantitative PCR assay for absolute quantification. (a) Melting curve, (b) linear regression equation. The amount of plasmid T-NP was converted into copy number via the following equation: copy number = (amount of T-NP × 6.022 × 10^23^)/(length of T-NP × 10^9^ × 660). The linearity of the standard curve was retained when the plasmid concentration was in the range of 5.5 × 10^2^–1.1 × 10^9^ copies/*μ*L. The obtained linear regression equation was *Y* = −3.2901*X* + 38.922 (*R* ^2^ = 0.998). The amplification efficiency was 101%.

DF1 cells were infected with La Sota at a MOI of 0.01, and the cells and supernatant were harvested at 48 hpi. The total RNAs were extracted and reverse-transcribed with primers Pla-G, Pla-R, and Pla-18V, respectively. We designed RT-primer Pla-G to bind to the leader sequence of gRNA, which is absent in cRNA and mRNA [24, 35]. RT-primer Pla-R was designed to bind to the P gene in front of the NP gene in viral antigenomic RNA; therefore, it is specific to viral cRNA. The PLa-18V primer is specific to all mRNA with a polyA tail, and viral gRNA and cRNA should not be reverse-transcribed. All the cDNAs of gRNA, cRNA, and mRNA were quantified with same qPCR primer pairs specific for the NP gene, Pla-rt13, and Pla-rt14, and similar amplification efficiency was observed in each reaction. The resultant cDNAs were tenfold serially diluted and detected using RT-qPCR. The *C*_*q*_ values of viral gRNA, cRNA, and NP gene mRNA ranged between 8.74–31.50, 8.76–31.46, and 9.26–32.12, respectively. The linear regression analysis revealed *R* ^2^ values in the range of 0.991–0.999 (Figure 2). The experiment was repeated three times and each sample was performed in triplicate. The coefficient of variation (CV) of *C*_*q*_ values ranged from 2.0% to 5.0% between each experiment and less than 0.5% between each repeat of sample.

### 3.2. Selection of the Incubation Time for La Sota Infection

**Figure 3:**
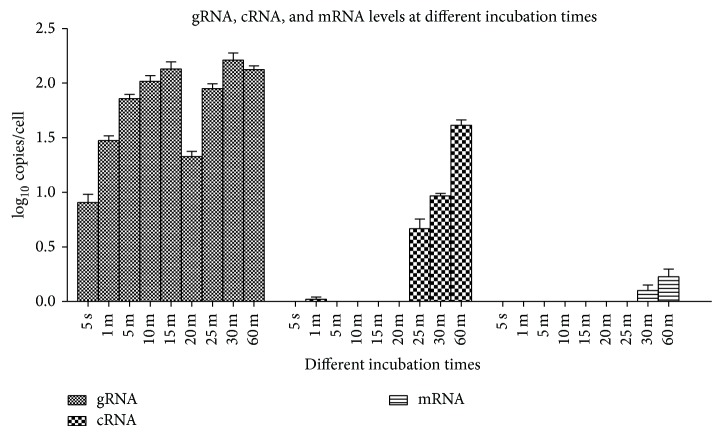
Selection of the optimal adsorption time for La Sota infection. gRNA, cRNA, and mRNA levels were measured at 5 s, 1 min, 5 min, 10 min, 15 min, 20 min, 25 min, and 30 min after DF-1 cells were infected with the La Sota strain at an MOI of 1. The optimal adsorption time was selected as 20 min, since the attachment and detachment of La Sota were balanced at this time point.

To investigate the interference from viral attachment and detachment, samples at different incubation times, ranging from 5 s to 60 min, were detected and the molecular numbers of intracellular RNA were quantified to determine RNA levels in the early phase of infection. The attachment and detachment during La Sota infection balanced 20 min after absorption (Figure 3). The viral RNA levels detected indicated the quantity of viral particles that entered cells by the adsorption time at the early phase of infection; the longer the adsorption time is, the higher the level of gRNA detected is. However, viral gRNA levels were low but still slightly higher than that at instantaneous adsorption (5 s), when the adsorption time was 20 min. The results suggest that invasion and detachment may be balanced at this time. In addition, there was no significant difference in viral gRNA levels between the adsorption times of 25, 30, and 60 min, and gRNA levels at these time points were higher than that at other time points. The viral cRNA became detectable at the adsorption time of 25 min and then increased rapidly, whereas the NP gene mRNA was detectable at the adsorption time of 30 min. Therefore, the incubation time for La Sota infection was determined as 20 min.

### 3.3. Intracellular Kinetics of Viral RNAs during NDV Infection

**Figure 4:**
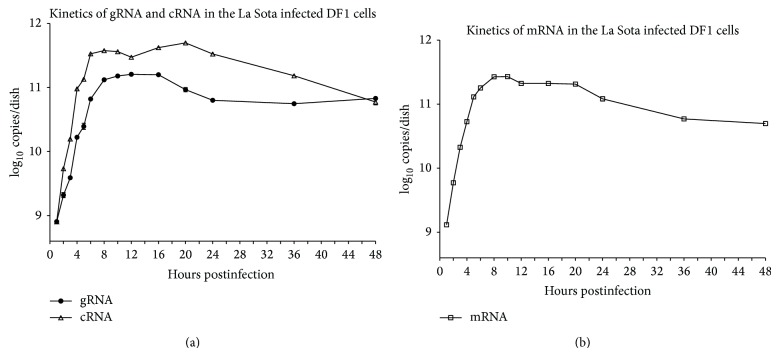
Kinetics of gRNA, cRNA, and mRNA in La Sota infected DF-1 cells. DF1 cells were incubated with NDV La Sota at an MOI of 1 for 20 min. The gRNA, cRNA, and mRNA were quantitatively analyzed at 1, 2, 3, 4, 5, 6, 8, 10, 12, 16, 20, 24, 36, and 48 hpi. (a) Kinetics of gRNA and cRNA. (b) Kinetics of NP gene mRNA.

The gRNA, cRNA, and viral mRNA levels had similar increasing trends in La Sota infected DF1 cells (Figure 4). In the earliest phase of NDV infection, viral RNA increased in a geometric progression; over the first 1 hpi, the gRNA copy number increased by more than one order of magnitude, from 1.2 × 10^6^ (the reference value at the adsorption time of 20 min) to 1.8 × 10^7^, whereas the cRNA and mRNA copy numbers increased from undetectable to almost the same level of gRNA (1.8 × 10^7^ for cRNA and 2.8 × 10^7^ for mRNA). Between 1 and 8 hpi, the viral RNA accumulated at an exponential rate, and gRNA, cRNA, and mRNA copy numbers increased by three orders of magnitude. The synthesis of mRNA peaked at 10 hpi and decreased sharply from 20 hpi. The synthesis of gRNA peaked at 12 hpi and declined from 16 hpi. The synthesis of cRNA peaked at 20 hpi, followed by its decrease. Throughout the course of infection, cRNA levels began to exceed gRNA levels since 1 hpi and subsequently remained at a level 1–5 times as high as that of the gRNA levels (Figure 4).

## 4. Discussion

NDV has a (−) single-stranded genomic RNA (gRNA), whereas the (+) single-stranded antigenomic RNA (cRNA) and mRNA are subsequently synthesized in cells upon NDV infection (Figure 1(a)). The genomic RNA of negative RNA viruses is a template for the synthesis of cRNA and mRNA [28, 36]. The role of nascent cRNA is to serve as a template for the synthesis of gRNA. Since no viral mRNA or protein is produced from cRNA, cRNA is considered an intermediate in the course of viral replication. A switching mechanism has been proposed in which viruses regulated the synthesis of different viral RNAs to facilitate infection. NP, M, and P proteins from influenza A virus, Sendai virus, and the measles virus have been reported to play an important role in the switching mechanism between transcription and replication [27, 29, 37–40]. However, a similar regulation of viral RNA synthesis was not explored in NDV until now.

To investigate the kinetics of viral transcription and replication in NDV-infected cells, a reliable real-time RT-PCR assay for the differential quantification of viral gRNA, cRNA, and mRNA was developed in this study. To simultaneously distinguish three kinds of viral RNA, three primers, PLa-G, PLa-R, and PLa-18V, specific for the reverse transcription of gRNA, cRNA, and mRNA, respectively, were designed and used for the assay. Due to different transcription efficiency between specific and random RT-primers, three kinetics curves for gRNA, cRNA, and mRNA were independently established to determine the synthesis pattern of these viral RNAs.

The relative molecular numbers of gRNA and cRNA in this study were comparable for at least three potential reasons. First, the sequences of the RT-primers, Pla-G, and Pla-R were exactly complementary to the target gene. Second, Pla-G and Pla-R were both located very close to the priming site of Pla-rt13 and Pla-rt-14 in the viral genome. Third, Pla-G and Pla-R displayed similar GC content.

In following specificity test, eight PCR fragments, Pla 1 to Pla 8, which covered most of the NDV genome except for the NP gene, were used for testing the specificity of primers. The results showed no cross-reaction with gene sequences other than NP, since their *C*_*q*_ values revealed levels far lower than that of the interest gene (T-NP), even lower than the testing limit. Therefore, by using a strand-specific real-time RT-PCR developed in this study, viral RNA accumulation kinetics in NDV-infected DF-1 cells were determined.

In our study, viral RNA synthesis was observed for the first time during the incubation of infection. The copy number of gRNA increased during incubation time, indicating an increase in the number of viruses entering cells with increasing incubation time. NDV cRNA was detectable at 25 min pi with a copy number of 8.7 per dish, and the NP mRNA was detectable at 30 min pi with copy numbers estimated at 0.5 per cell (Figure 3), results meeting the requirement for NDV infection and spread. Interestingly, trace viral cRNA was detected after 5 min of incubation and then detected when incubated for more than 25 min; in comparison, no viral mRNA was detected with incubations less than 30 min. This result appears to contradict reports that viral mRNA synthesis precedes cRNA synthesis in the early phase of infection [30–32, 41]. However, these reports were based on data detected after incubation. In this study, the results displayed the unstable state of nascent viral RNA at the early times of infection.

Based on our results, viral cRNA and mRNA can hardly be detected in the initial 25 min after virus-cell contact (Figure 3). It is reasonable to infer that those viral RNAs were degraded at the every early time of infection, similar to that of influenza virus cRNA and mRNA [32]. First of all, viral mRNA will be degraded via basic mRNA decay machinery or antiviral mRNA turnover system [42–44]. Secondly, naked gRNA or cRNA chain will be degraded by cellular nucleases unless covered by NP proteins following the rule of six [10, 24]. In addition, NDV replication and transcription depend on the encapsidation of gRNA and cRNA. Therefore, NP proteins should be preferentially synthesized, and NP gene mRNA transcripts should be abundantly generated at the earliest period of infection [36]. Viral cRNA and mRNA would be steadily detected until their synthesis rates are higher than degradation rates.

After an incubation of 25 min, the gRNA level increased rapidly. During the first 6 hpi, the levels of gRNA, cRNA, and mRNA were always in a phase of exponential increase, potentially related to the acute infection characteristics of NDV. The kinetics of NDV gRNA, cRNA, and mRNA synthesis in the infected DF-1 cells were shown to be consistent and could be divided into three phases: between 1 and 8 hpi, when viral RNAs accumulated exponentially (Phase I), followed by a plateau (Phase II), followed by a decrease between 16 and 20 hpi (Phase III). Neither NDV transcription nor replication was predominant in the earliest phase of NDV infection.

The replication of NDV involves the synthesis of gRNA and cRNA. In this study, cRNA was synthesized faster than gRNA (Figure 4). Our results showed that at 1 hpi, cRNA synthesis was initiated and its amount rapidly exceeding that of gRNA. The cRNA levels were maintained 3–5 times higher than that of gRNA until 48 hpi. In other words, the amount of cRNA was higher than that of gRNA altogether within infected cells and supernatant. Theoretically, the amount of gRNA would be higher than that of cRNA, because only gRNA (a template for mRNA and cRNA) is assembled into viral particles and released out of the cells. The rapid accumulation of cRNA versus gRNA could be a specific characteristic of NDV or paramyxoviruses. Similar results were reported in the Nipah virus [32], where the amount of intracellular vRNA of Nipah was much higher than extracellular vRNA. It was believed that Nipah virus particles were not immediately released into the cell culture medium. However, the proportion of cRNA in the intracellular vRNA was not determined in the study. Our results here suggest that cRNA can be even higher than vRNA altogether in infected cells and supernatant.

To understand the above results, the outcome of gRNA in viral particles released in supernatant should be reconsidered. Most gRNAs are assembled into viral particles that bud into cultural supernatant. However, those progeny viruses in supernatant actually gradually degrade under an environment temperature of 37°C unless they infect cells again [45–48]. The gRNA also decays following the death of viral particles. In contrast, cRNA may behave differently. As a paramyxovirus, NDV cRNA is totally encapsidated by the NP protein that protects cRNA from degradation by cellular nucleases. It is reasonable to infer that most cRNA is preserved in cells and accumulates steadily. To conclude, a partially degradation of gRNA and preservation of cRNA would explain why the amount of progeny cRNA was higher than that of gRNA in our study.

## 5. Conclusion

To conclude, a real-time RT-PCR assay for absolute quantitation of specific viral RNA fragments in La Sota infected cells was developed for the first time. With this method, we revealed that the cRNA of NDV accumulated faster than its vRNA. The development of this assay will be helpful for further studies on the pathogenesis and control strategies of NDV.

## Figures and Tables

**Table 1:** Primers used in this study.

Primers	Sequences (5′-3′)	Location^3^	Fragment designation
Pla1F^1^	TATCCAGGCTCAAGTATGGGTCACA	622–646	Pla1
Pla1R^2^	CCTTGGTCTTGCCTTGTGGGATTG	1999–2022	
Pla2F	ATATTCAGAGATCAGGGCAAGTC	1819–1841	Pla2
Pla2R	GTTTGCCACAACCCTACAGC	3708–3724	
Pla3F	AAAGCTGTAGGGTTGTGG	3705–3727	Pla3
Pla3R	TTGGCGATGACTGAACCT	5704–5721	
Pla4F	AGGCGCACTTACTACACCATA	5671–5691	Pla4
Pla4R	TGCTGTATGCGTTTCCCACCA	7502–7525	
Pla5F	TACAGCAGGCTATCTTATCT	7517–7536	Pla5
Pla5R	TGAGTCACGGATTCTGCT	9370–9387	
Pla6F	GTGACTCATGCAATCGCTACT	9377–9400	Pla6
Pla6R	CTAATTGGGCAGGAGTCAGA	11106–11126	
Pla7F	TGCAGAGATCAAGCGACTA	11197–11215	Pla7
Pla7R	AGCATCTTCTCATTCAGGTTATC	13105–13128	
Pla8F	TATCCCGGTTATGCTGTC	13137–13154	Pla8
Pla8R	TAAGACATTTATTTGAGTTCG	15103–15122	
Pla-rt11	CAATAGGAGTGGAGTGTCTGA	466–486	Real time
Pla-rt12	TCCTCTCCAGGGTATCGGTGA	549–614	
Pla-rt13	CAACAATAGGAGTGGAGTGTCTGA	463–486	Real time
Pla-rt14	CAGGGTATCGGTGATGTCTTCT	586–607	
Pla-rt21	TCTGTACTGTACTTGACTCGTGCTC	14903–14927	Real time
Pla-rt22	CTGTAATATCCGTTGACTGCATTGCCT	14953–14979	

^1^F represents forward primer.

^2^R represents reverse transcription- (RT-) primer.

^3^The location was based on the genomic sequence of NDV La Sota (GenBank ID: LaSota_AF077761).

## Abbreviations

$C_{q}$: Quantitation cycle
cRNA: Antigenomic RNA
CV: Coefficient of variation
F: Fusion protein
gRNA: Genomic RNA
HN: Haemagglutinin-neuraminidase
L: Large protein
M: Matrix protein
MOI: Multiplicity of infection
mRNA: Messenger RNA
ND: Newcastle disease
NDV: Newcastle disease virus
NP: Nucleoprotein
P: Phosphoprotein
PBS: Phosphate-buffered saline
pi: postinfection
qPCR: Quantitative PCR
RT: Reverse transcription
RT-PCR: Reverse transcription polymerase chain reaction
SPF: Specific pathogen-free
TCID_50_: 50% tissue culture infection dose
vRdRp: Viral RNA-dependent RNA polymerase
vRNA: Viral RNA.
